# Development of an interpretable machine learning model to predict complete remission and first adverse event in pediatric acute myeloid leukemia using routine clinical data

**DOI:** 10.3389/fonc.2026.1739432

**Published:** 2026-04-10

**Authors:** Ningshu Huang, Weiwei Wang, Rui Yang, Zhihong Liao, Hui Yang, Xi Chen

**Affiliations:** 1 Department of Clinical Laboratory, Children’s Hospital of Chongqing Medical University, National Clinical Research Center for Children and Adolescents' Health and Diseases, Ministry of Education Key Laboratory of Child Development and Disorders, Chongqing Key Laboratory of Pediatrics Metabolism and Inflammatory Diseases, Laboratory for clinical diagnostic and translational research of CHCQMU, Chongqing, China; 2 Department of the Clinical molecular Center, Children's Hospital of Chongqing Medical University, National Clinical Research Center for Children and Adolescents' Health and Disorders, Ministry of Education Key Laboratory of Child Development and Disorders, Chongqing Key Laboratory of Pediatrics Metabolism and Inflammatory Diseases, Laboratory for clinical diagnostic and translational research of CHCQMU, Chongqing, China; 3 Department of Hematology, Children’s Hospital of Chongqing Medical University, National Clinical Research Center for Children and Adolescents' Health and Disorders, Ministry of Education Key Laboratory of Child Development and Disorders, Chongqing Key Laboratory of Pediatrics Metabolism and Inflammatory Diseases, Chongqing, China

**Keywords:** adverse event, complete remission, interpretablemachine learning, pediatric acute myeloid leukemia, SHAP analysis

## Abstract

**Background:**

Pediatric acute myeloid leukemia (pAML) is a rapidly progressive myeloid malignancy characterized by malignant clonal expansion of hematopoietic stem and progenitor cells. The prediction of complete remission (CR) and first adverse event (AE) is critical for personalizing pAML treatment; however, interpretable machine learning (ML) models that utilize only routine clinical features for this purpose are lacking.

**Methods:**

A total of 206 *de novo* pediatric AML patients (excluding acute promyelocytic leukemia) were randomly split into training (80%) and test (20%) sets. Seven supervised ML algorithms were constructed for predicting CR and AE, and their performance was evaluated by accuracy, specificity, F1-score, and area under the receiver operating characteristic curve (AUC). Model interpretability was assessed using feature importance, accumulated local effect (ALE) plots, and SHAP values.

**Results:**

To identify optimal predictors of CR, three feature selection methods-random forest, stepwise regression, and joint mutual information maximization (JMIM)-were employed. Their intersection revealed seven key features: age, bone marrow blasts, peripheral blood blasts, platelet count (PLT), t (8;21), TP53 and del7/del7q. The random forest model demonstrated optimal performance, with a training AUC of 0.90 (95% CI: 0.86-0.97) and a test AUC of 0.79 (95% CI: 0.72-0.86). The similar machine learning pipeline was applied to predict the first adverse event (AE). Nine features were selected as optimal predictors through the intersection of the same three algorithms: white blood cell count (WBC), peripheral blood blasts, PLT, bone marrow blasts, hemoglobin, age, t (8;21), NPM1 and KIT. For AE prediction, the random forest algorithm also exhibited optimal performance, with a training AUC of 0.92 (95% CI: 0.85-0.97) and a test AUC of 0.78 (95% CI: 0.66-0.84). Interpretability analysis of the random forest models revealed that a higher platelet count at diagnosis was predictive of an increased probability of CR and a reduced risk of AE. In contrast, elevated WBC and peripheral blood blast percentage were associated with a higher incidence of AE.

**Conclusion:**

Our random forest model, built on routine hematological parameters, demonstrated strong potential for predicting CR and AE in pAML, thereby facilitating early risk stratification and guiding personalized treatment strategies.

## 1 Introduction

Pediatric acute myeloid leukemia (pAML) is a rapidly progressing myeloproliferative disorder arising from the malignant clonal proliferation of hematopoietic stem/progenitor cells (1). Because of its highly heterogenity, the therapeutic advancements for pAML remain significantly behind those of other leukemic subtypes, and the 10-year overall survival rate for affected children remains below 50% (2). The primary goal of initial therapy for pAML is to achieve complete remission (CR) (3). Nevertheless, 10-20% of patients fail to achieve CR after initial induction (4), frequently attributed to factors like a high baseline leukemic cell burden or unfavorable genetic profiles. Allogeneic hematopoietic stem cell transplantation (allo-HSCT) is typically indicated for pAML patients with high-risk pathologic features or refractory AML (5). Nevertheless, the survival advantage of allo-HSCT must be weighed against transplantation-related mortality (TRM) and long-term adverse events, such as graft-versus-host disease (GVHD) (6). Approximately 30% of pAML patients experience relapse, and prognosis for relapsed patients remains dismal-particularly among those with adverse risk profiles or prior allo-HSCT exposure (7). Thus, high relapse rates and refractory cases continue to pose formidable challenges in pAML management (8).

Against this backdrop, accurate diagnosis and effective treatment of pAML have always been the focus of pediatric hematology and oncology (9–11). Nowadays, pAML diagnosis relies on a multi-modality diagnostic framework incorporating cytomorphology, immunophenotyping, cytogenetics, and molecular genetic testing (7). Among these modalities, cytogenetic and molecular profiles act as pivotal determinants for prognosis assessment and risk stratification, while also guiding the formulation of personalized therapeutic protocols-both critical to enhancing survival outcomes and quality of life in affected children (7, 11). However, with the in-depth development of genomic research, the volume of genomic data related to pAML has expanded rapidly (12–15), bringing enormous challenges to the effective translation of these data into clinical practice and the optimization of clinical decision-making. Machine learning (ML) has emerged as a powerful tool in the field of biomedical research (16). Due to its outstanding ability to process complex and high-dimensional biomedical datasets (17–19), ML has shown great application potential in promoting the development of precision medicine and optimizing leukemia management strategies.

**Figure 1:**
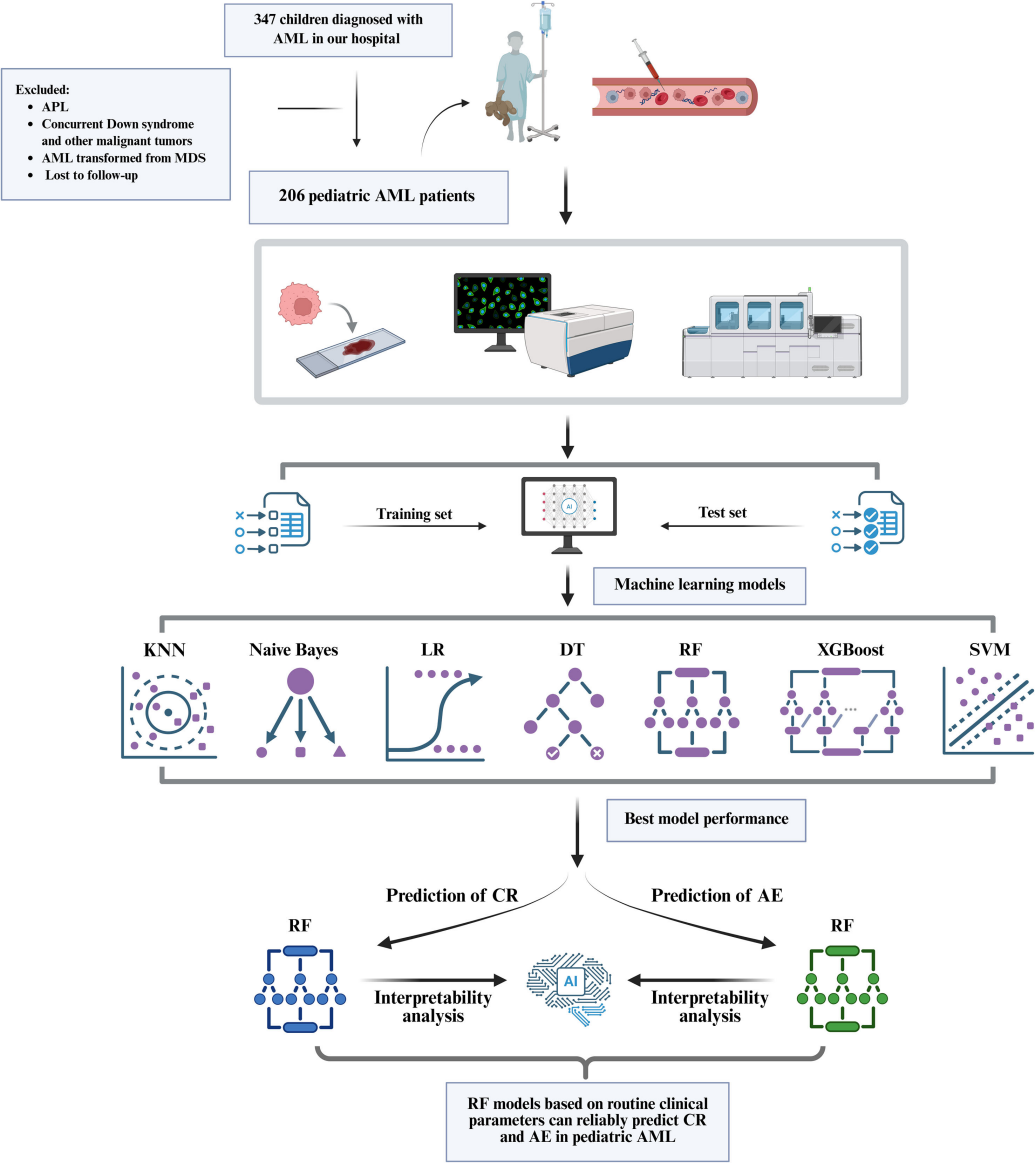
Flowchart of the study. Created with BioRender.com. APL, acute promyelocytic leukemia; AML, acute myeloid leukemia; MDS, myelodysplastic syndrome; KNN, k nearest neighbor; LR, logistic regression; DT, decision tree; RF, random forest; SVM, support vector machine; CR, complete remission; AE, adverse event.

Despite the promising prospects of ML in pAML research, a notable gap exists in the current research: there are no previous studies that have developed machine learning models only using routine clinical parameters to predict complete remission (CR) or first adverse event (AE; defined as those events such as failure to achieve complete remission, disease relapse, or death from any cause). To fill this research gap, our study constructed seven machine learning algorithms-including k nearest neighbor (KNN), decision tree (DT), random forest (RF), XGBoost, support vector machine (SVM), logistic regression (LR), and Naive Bayes-using the clinical data of 206 children diagnosed with AML (excluding acute promyelocytic leukemia) at our hospital over a 10-year period. Preliminary results of our analysis indicated that these ML models, especially the random forest (RF) algorithm, achieved excellent predictive performance for both CR (AUC 0.90) and AE (AUC 0.92) (**Figure 1**). This study aims to provide a reliable and convenient predictive tool for clinical practice, thereby assisting clinicians in making more accurate treatment decisions and improving the clinical outcomes of pediatric AML patients.

## 2 Materials and methods

### 2.1 Research subjects

This retrospective study was conducted at Children’s Hospital of Chongqing Medical University from July 2008 to October 2018. There were 347 children diagnosed with AML in our hospital and classified according to the 2008 (20) or 2016 World Health Organization (WHO) (21) classification, respectively. The inclusion criteria included patients diagnosed with *de novo* AML, aged under 18 years, and accepting regular and adequate treatment. Pediatric patients with acute promyelocytic leukemia (APL), concurrent Down syndrome and other malignant tumors, AML transformed from myelodysplastic syndrome (MDS), and patients lost to follow-up were excluded. In total, 206 pediatric AML patients met the eligibility criteria for the review. The approval for our research was obtained from the Children’s Hospital of Chongqing Medical University Institutional Review Board (2024–134). All information was collected anonymously, and the requirement for informed consent was waived.

### 2.2 Date set

We collected medical records of enrolled children, including demographic data, clinical variables, treatment, and outcomes. The treatment plans for pediatric AML patients were according to the guidelines of the Hematology Group of the Pediatrics Branch of the Chinese Medical Association in 2006 (AML-2006) (22)(**Supplementary Figure 1**) and the 2015-AML-03 protocol developed by our hospital (23) (**Supplementary Figure 2**), which was based on AML-2006. Based on the treatment protocol in effect at that time, 129 patients received the AML-2006 regimen, and 77 received the 2015-AML-03 regimen. Complete remission (CR) was defined as the recovery of normal hematopoiesis in the bone marrow, less than 5% blasts in bone marrow, no blasts with Auer rods, absence of extramedullary disease, absolute neutrophil count (ANC) > 1.0×10^9^/L, platelet count > 80.0×10^9^/L, and independence from transfusing red cells (24). Event-free survival (EFS) was defined as the time from children’s diagnosis of AML to the occurrence of the first adverse event, which includes three components: (1) failure to achieve remission, (2) disease relapse, and (3) death resulting from any cause. In our study, the first adverse event (AE) was defined as a composite endpoint because each component represents a clinically severe outcome. In addition, given the limited sample size and number of individual component events, using a composite endpoint increased the event rate and statistical power for developing the prediction model designed to identify the pediatric AML patients at high risk for any major adverse outcome during the disease course.

### 2.3 Machine learning model design and performance evaluation

Seven different supervised machine learning algorithms were used in this research, including k nearest neighbor (KNN), decision tree (DT), random forest (RF), XGBoost, support vector machine (SVM), logistic regression (LR) and Naive Bayes. Machine learning models were performed by R package mlr3. The dataset was stratified into two subgroups based on the treatment protocol received: AML-2006 and 2015-AML-03. We performed independent feature selection within each subgroup using CR or AE as the outcome, with three methods applied in each case: random forest, stepwise regression, and joint mutual information maximization (JMIM). This generated a total of six separate candidate feature lists. The intersection of all six lists was taken to determine the optimal feature variables for the machine learning algorithms. Specifically, the random forest method identified the top 20 important variables based on the contribution of Increase in Node Purity (IncNodePurity). Additionally, the JMIM filter feature selection algorithm selected the top 10 features based on the amount of information they provided. The grid search algorithm with 10-fold cross-validation was used to tune hyperparameters for models based on classification error. The 206 patients were randomly divided into training and test sets (8:2 ratio), with all feature selection and hyperparameter tuning strictly confined to the training set. The performance of different models was compared based on the confusion matrix, from which metrics such as accuracy, specificity, F1-score, receiver operating characteristic (ROC) curve, and the area under the curve (AUC) were calculated. These steps were performed using R 4.2.2, and the R package mlr3 (Version 0.16.1), mlr3learners (Version 0.5.6), mlr3tuning (Version 0.19.0), mlr3viz (Version 0.6.1), randomForest (Version 4.7-1.1) and ggplot2 (Version 3.4.3).

### 2.4 Statistical analysis

The descriptive statistics of continuous variables were expressed as median and interquartile range and categorical variables were presented using frequency and percentage. The bootstrap method with 1000 iterations was applied to the internal validation set to estimate the confidence intervals for AUROC, accuracy, specificity, and F1-score. The R package boot (Version 1.3-28.1) and mlr3measures (Version 0.5.0) were used to perform these calculations.

### 2.5 Model interpretation

The R package iml was employed to interpret the behavior of the machine learning models. In order to apply the interpretation methods provided by iml, the models and data were wrapped in the “Predictor” object. Furthermore, the “FeatureImp” interpretation method was selected, which computes the importance of features by calculating the increase in the model’s prediction error after permuting the feature. Subsequently, the Feature Effects were showed by the Accumulated Local Effect (ALE) plots. Finally, to explain individual predictions, SHAP (Shapley Additive Explanations) values were computed and visualized, illustrating the contribution of each feature to a single prediction outcome, thereby facilitating a transparent understanding of the model’s decision-making process.

## 3 Results

### 3.1 Pediatric patient characteristics

We constructed seven machine learning models to predict complete remission (CR) and first adverse event, such as death or recurrent, in our data set of 206 (110 male and 96 female) newly diagnosed pediatric AML patients with the median age of 6.64 years (**Table 1**). For French-American-British (FAB) classification, pediatric patients in the data set were of seven sub-type AML excepting M3 acute promyelocytic leukemia (APL), including M0 (2.42%), M1 (7.77%), M2 (45.63%), M4 (7.28%), M5 (25.73%), M6 (4.37%), and M7 (6.80%) (**Table 1**). Based on the molecular/cytogenetic features and the response to induction chemotherapy, as described in Children’s Oncology Group reports (25), patients were divided into three risk stratifications: favorable (37.86%), intermediate (52.91%), and adverse (9.22%) (**Table 1**). As shown in **Table 1**, a high incidence of abnormal karyotype (69.90%) was detected in pediatric patients with AML. Furthermore, complex karyotype (≥ 3 abnormalities) was identified in 23 patients (11.17%). Cytogenetic and molecular features played an important role in the classification of pediatric AML according to the WHO guidelines. Detailed information regarding these characteristics of patients in our data set was summarized in **Supplementary Table 1**. In pediatric AML, the most common symptoms are typically related to leukemia burden and the reduction of blood cells. In our study, we observed a significant increase in the proportion of primitive cells, with median values of 42% in peripheral blood and 66% in bone marrow (**Table 1**). Moreover, the decrease in blood cells can lead to conditions such as anemia, thrombocytopenia and neutropenia. Specifically, the median value of hemoglobin was 74g/L, and the median value of platelet count (PLT) was 37.5×10^9^/L in our research (**Table 1**). A total of 142 patients (68.93%) achieved CR after induction therapy, while 64 (31.07%) patients failed to achieve CR. The median event-free survival (EFS) was 12.40 months in our data set (**Table 1**).

**Table 1:** Clinical characteristics (N = 206 patients).

Varialbles	Data
Age, median (IQR), year	6.64 (0.42- 10.00)
Sex, male, N (%)	110 (53.40)
FAB classification, N (%)	
M0	5 (2.42)
M1	16 (7.77)
M2	94 (45.63)
M4	15 (7.28)
M5	53 (25.73)
M6	9 (4.37)
M7	14 (6.80)
COG Risk Classification 2017, N (%)	
Favorable	78 (37.86)
Intermediate	109 (52.91)
Adverse	19 (9.22)
Abnormal karyotype, N (%)	144 (69.90)
Complex karyotype (≥ 3 abnormalities), N (%)	23 (11.17)
WBC, median (IQR), ×10^9^/L	17.16 (5.84-48.92)
Hb, median (IQR), g/L	74.00 (60.00-84.75)
PLT, median (IQR), ×10^9^/L	37.50 (19.00-69.75)
Peripheral blood blasts, median (IQR), %	42.00 (19.25- 69.00)
Bone marrow blasts, median (IQR), %	66.00 (42.00-80.00)
Achieved CR after induction therapy, N (%)	142 (68.93)
median EFS (IQR), month	12.40 (5.95-37.48)

FAB, French-American-British; COG, Children’s Oncology Group; WBC, white blood cells; Hb, Hemoglobin; PLT, platelet; CR, complete remission; EFS, event-free survival IQR, interquartile range.

### 3.2 Prediction of complete remission by machine learning models

**Figure 2:**
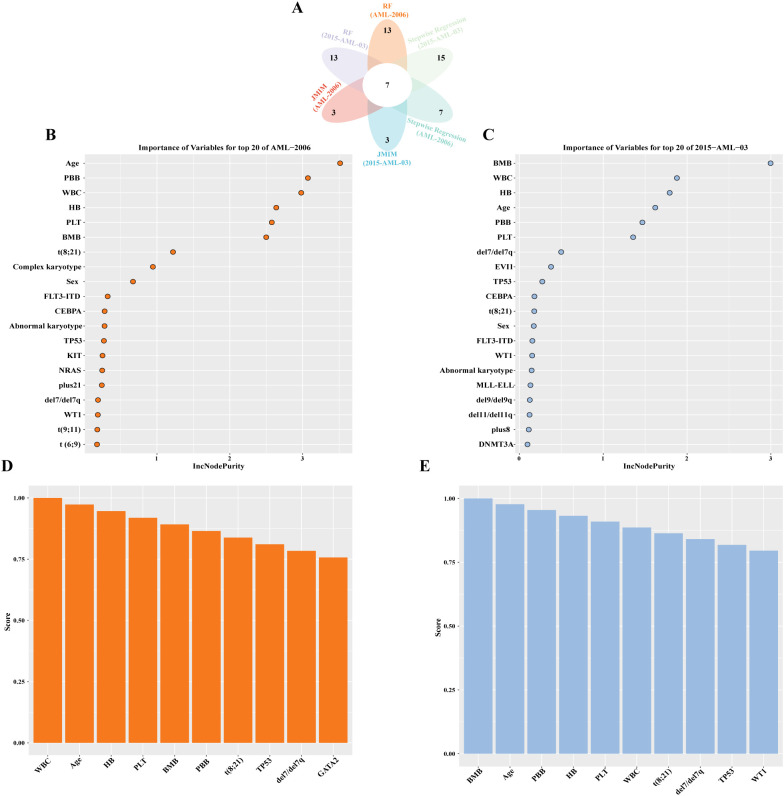
Identification of core predictive features for complete remission (CR) through three algorithm selection within treatment subgroups. **(A)** Venn intersection analysis of the algorithm outputs, ultimately identifying seven core feature variables for predicting CR. **(B)** Results of random forest for AML-2006 treatment subgroup. **(C)** Results of random forest for AML-2015–03 treatment subgroup. **(D)** Results of joint mutual information maximization (JMIM) for AML-2006 treatment subgroup. **(E)** Results of joint mutual information maximization (JMIM) for AML-2015–03 treatment subgroup.

**Figure 3:**
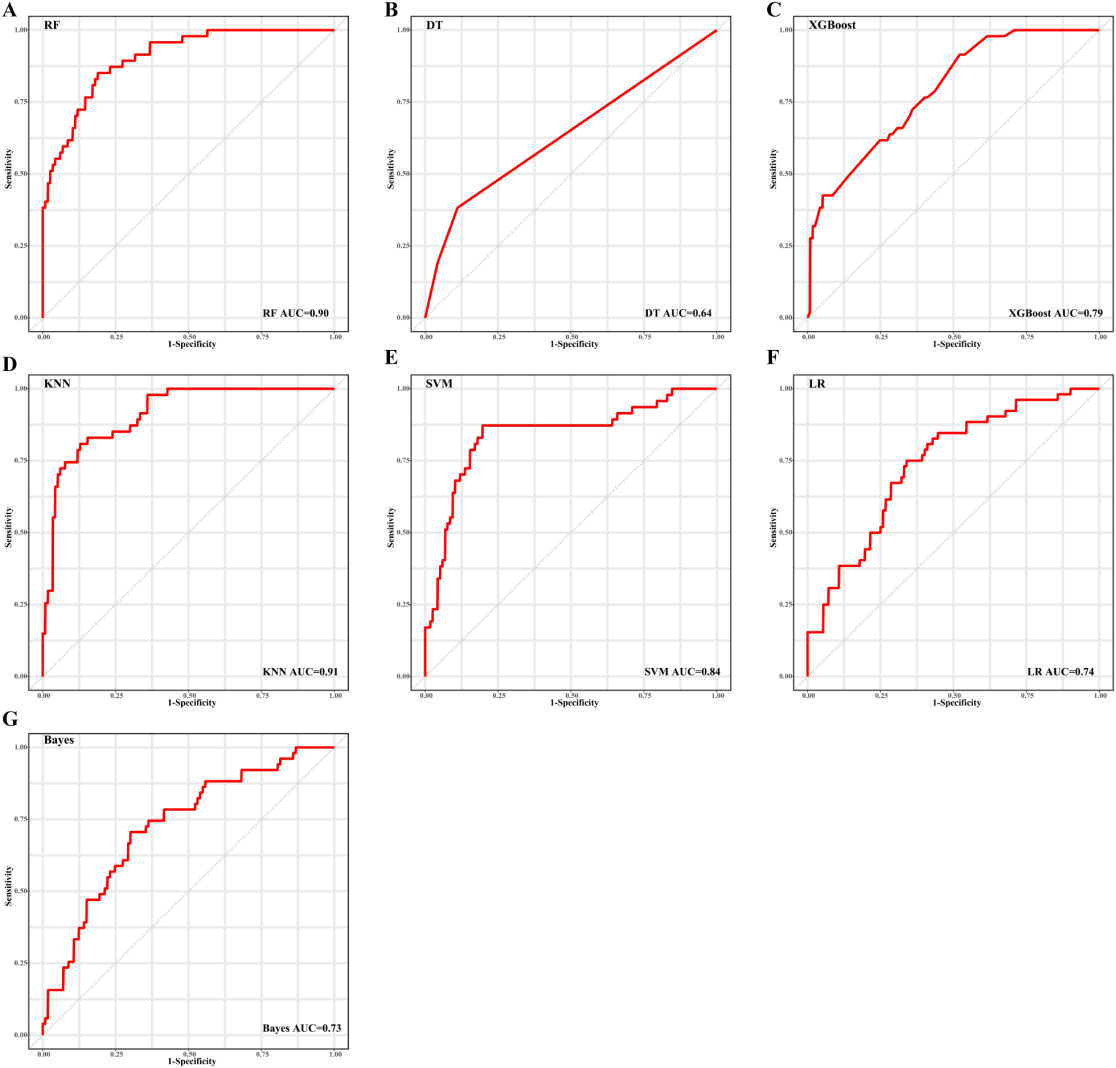
Performance of seven machine learning algorithms for CR prediction, as shown by area under the receiver operating characteristic curve (AUROC) results in the training set. **(A)** random forest (RF), **(B)** decision tree (DT), **(C)** XGBoost, **(D)** k nearest neighbor (KNN), **(E)** support vector machine (SVM), **(F)** logistic regression (LR), and **(G)** Naive Bayes.

To identify the optimal feature variables for predicting CR, three feature selection algorithms were employed: random forest (**Supplementary Figure S3**), stepwise regression, and joint mutual information maximization (JMIM) (**Figures 2B–E**). The intersection of these algorithms resulted in the selection of seven features (age, bone marrow blasts, peripheral blood blasts, PLT, t (8,21), TP53 and del7/del7q) (**Figure 2A**). These seven selected features were utilized to build seven machine learning models for CR prediction. The models included k nearest neighbor (KNN), Naive Bayes, logistic regression (LR), decision tree, random forest (RF), XGBoost, and support vector machine (SVM). In addition to feature selection, tuning the hyperparameters of the models was essential to achieve optimal performance on our datasets (**Supplementary Table 2**). The area under the receiver operating characteristics curve (AUROC) for predicting complete remission ranged from 0.64 to 0.91 in the train set (**Figure 3**) and between 0.59 and 0.79 in the test set (**Supplementary Figure 4**) across the different models. The models built using Naive Bayes, LR, RF and XGBoost algorithms all demonstrated robust predictive accuracy. The AUC values for predicting CR in pediatric AML patients exceeded 0.7 in both the training and testing sets. Notably, the Random Forest model demonstrated robust performance, achieving an AUC >0.75 and a Brier score <0.20 across both the training (AUC: 0.90, Brier score: 0.16) and test (AUC: 0.79, Brier score: 0.19) sets. To further evaluate the performance of each model, additional metrics were assessed and shown in **Table 2**. The results indicated that the RF model displayed excellent accuracy (train set: 0.86, 95% CI 0.80-0.91; test set: 0.76, 95% CI 0.70-0.88), specificity (train set: 0.99, 95% CI 0.97-1.00; test set: 0.91, 95% CI 0.81-1.00) and F1-score (train set: 0.90, 95% CI 0.86-0.94; test set: 0.86, 95% CI 0.76-0.94) in predicting complete remission for pediatric AML patients.

**Table 2:** Model performance for prediction of CR.

Model		Accuracy (95%CI)	Specificity (95%CI)	F1 score (95%CI)	AUROC (95%CI)
KNN	Train	0.80 (0.74, 0.86)	0.97 (0.92, 0.99)	0.88 (0.83, 0.91)	0.91 (0.83, 0.92)
	Test	0.64 (0.50, 0.79)	0.92 (0.80, 1.00)	0.75 (0.62, 0.87)	0.63 (0.58, 0.87)
Naive Bayes	Train	0.70 (0.62, 0.77)	0.94 (0.89, 0.98)	0.81 (0.75, 0.86)	0.73 (0.65, 0.81)
	Test	0.74 (0.60, 0.86)	0.97 (0.89, 1.00)	0.84 (0.73, 0.92)	0.71 (0.63, 0.82)
LR	Train	0.71 (0.64, 0.77)	0.89 (0.83, 0.94)	0.81 (0.78, 0.88)	0.74 (0.65, 0.86)
	Test	0.74 (0.60, 0.86)	0.97 (0.88, 1.00)	0.84 (0.73, 0.92)	0.72 (0.63, 0.82)
Decision Tree	Train	0.74 (0.68, 0.81)	0.89 (0.83, 0.94)	0.83 (0.78, 0.88)	0.64 (0.58, 0.68)
	Test	0.64 (0.50, 0.78)	0.84 (0.68, 0.96)	0.74 (0.58, 0.85)	0.60 (0.58, 0.86)
RF	Train	0.86 (0.80, 0.91)	0.99 (0.97, 1.00)	0.90 (0.86, 0.94)	0.90 (0.86, 0.97)
	Test	0.76 (0.70, 0.88)	0.91 (0.81, 1.00)	0.86 (0.76, 0.94)	0.79 (0.72, 0.86)
XGBoost	Train	0.79 (0.73, 0.85)	0.97 (0.95, 1.00)	0.87 (0.82, 0.91)	0.79 (0.62, 0.81)
	Test	0.74 (0.60, 0.86)	0.92 (0.80, 1.00)	0.81 (0.67, 0.90)	0.74 (0.67, 0.80)
SVM	Train	0.76 (0.69, 0.82)	0.99 (0.97, 1.00)	0.85 (0.81, 0.90)	0.84 (0.81, 0.90)
	Test	0.60 (0.57, 0.74)	0.90 (0.80, 0.98)	0.75 (0.62, 0.85)	0.59 (0.52, 0.65)

CR, complete remission; KNN, k nearest neighbor; LR, logistic regression; RF, random forest; SVM, support vector machine; AUROC, area under the receiver operating characteristics curve.

### 3.3 Prediction of adverse event by machine learning models

**Figure 4:**
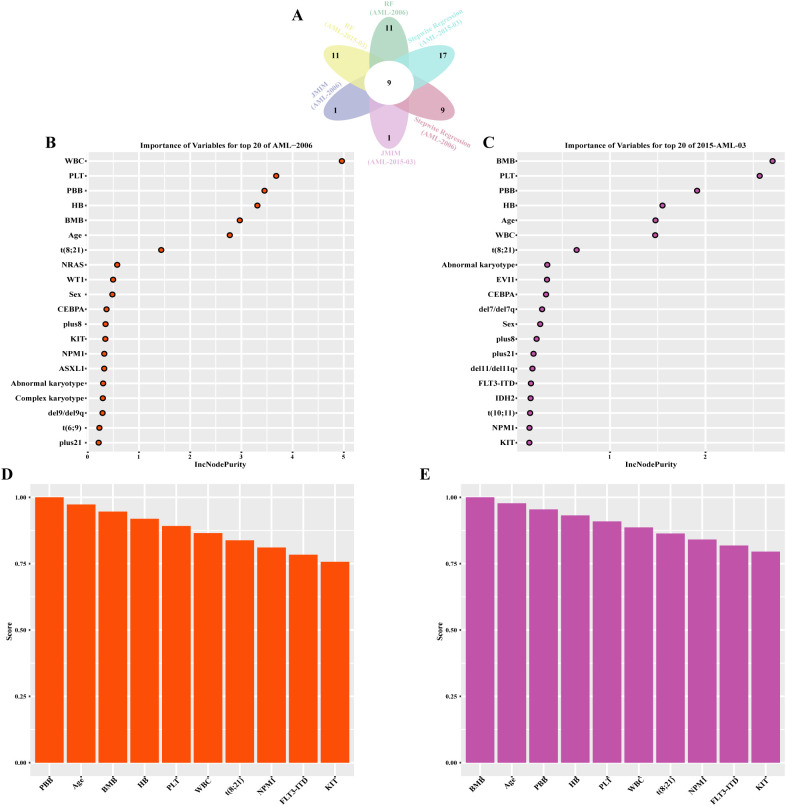
Identification of core predictive features for first adverse event (AE) through three algorithm selection within treatment subgroups. **(A)** Venn intersection analysis of the algorithm outputs, ultimately identifying nine core feature variables for predicting AE. **(B)** Results of random forest for AML-2006 treatment subgroup. **(C)** Results of random forest for AML-2015–03 treatment subgroup. **(D)** Results of joint mutual information maximization (JMIM) for AML-2006 treatment subgroup. **(E)** Results of joint mutual information maximization (JMIM) for AML-2015–03 treatment subgroup.

**Figure 5:**
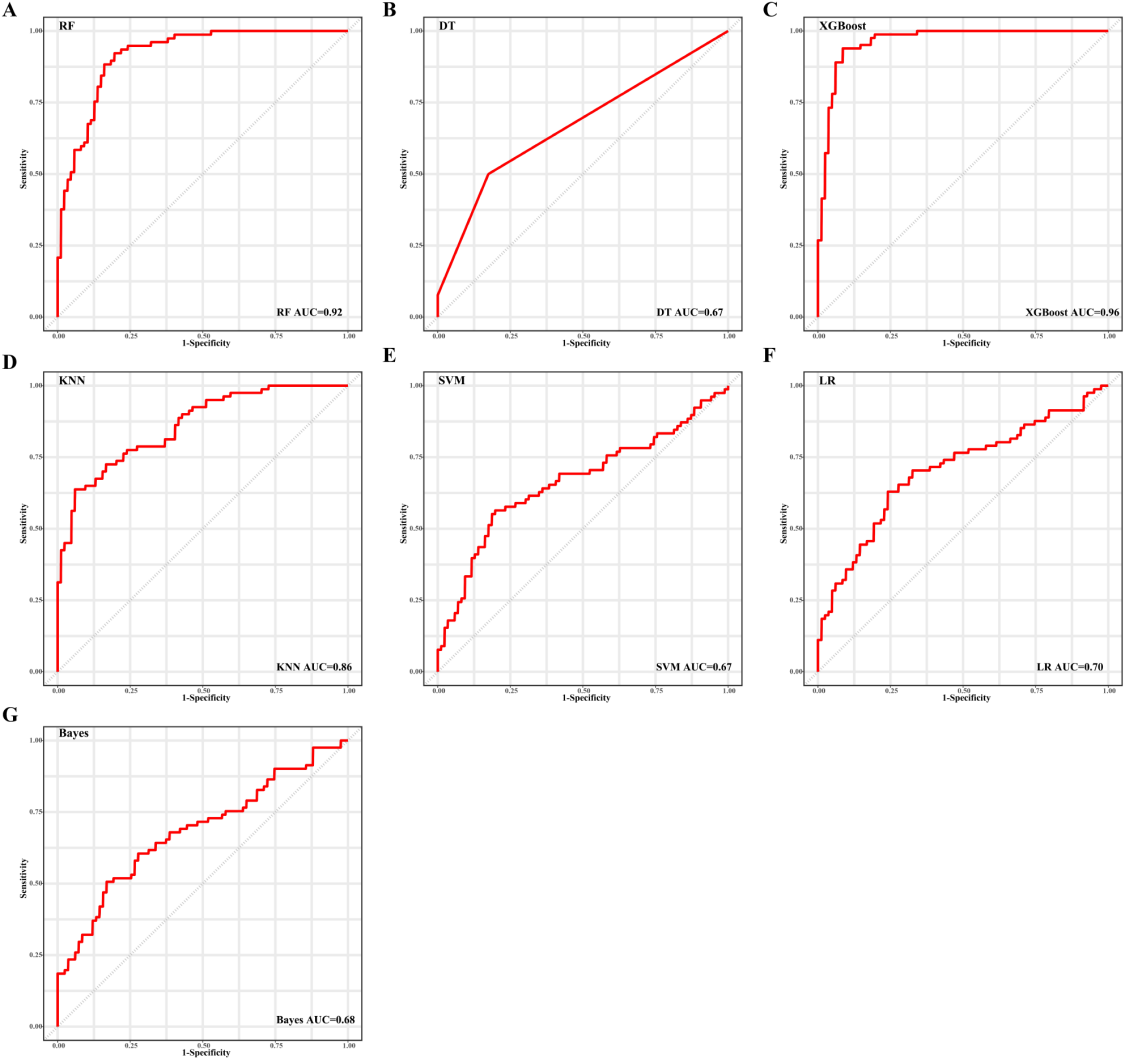
Performance of seven machine learning algorithms for AE prediction, as shown by area under the receiver operating characteristic curve (AUROC) results in the training set. **(A)** random forest (RF), **(B)** decision tree (DT), **(C)** XGBoost, **(D)** k nearest neighbor (KNN), **(E)** support vector machine (SVM), **(F)** logistic regression (LR), and **(G)** Naive Bayes.

Similar to the prediction of CR, the machine learning pipeline was used to predict the occurrence of the first adverse event (AE). As shown in **Figure 4**, nine features were selected as optimal variables through the intersection of random forest (**Supplementary Figure 5**), stepwise regression, and JMIM. These features included WBC, peripheral blood blasts, PLT, bone marrow blasts, hemoglobin, age, t (8;21), NPM1 and KIT. Again, the hyperparameters of KNN, decision tree, RF, XGBoost, and SVM were fine-tuned using the R package mlr3 (**Supplementary Table 3**). The performance of the seven machine learning algorithms for predicting AE showed promise, with AUROC values of 0.86, 0.68, 0.70, 0.67, 0.92, 0.96, and 0.67 in the training set (**Figure 5**), and 0.61, 0.68, 0.66, 0.61, 0.78, 0.65, and 0.63 in the testing set (**Supplementary Figure 6**) for KNN, Naive Bayes, LR, Decision Tree, RF, XGBoost, and SVM, respectively. Among these models, RF exhibited the best performance for predicting the occurrence of the first adverse event, achieving an AUROC of 0.92 and a Brier score of 0.18 in the training cohort, while maintaining an AUROC of 0.78 and a Brier score of 0.20 in the test cohort. Additionally, in the training set, the RF model demonstrated an accuracy of 0.78 (95% CI 0.72-0.84), specificity of 0.90 (95% CI 0.83-0.96), and F1-score of 0.81 (95% CI 0.75-0.87). In the testing set, RF showed values of 0.70 (95% CI 0.62-0.74) for accuracy, 0.95 (95% CI 0.82-1.00) for specificity, and 0.72 (95% CI 0.68-0.84) for F1-score (**Table 3**).

**Table 3:** Model performance for prediction of first adverse event.

Model		Accuracy (95%CI)	Specificity (95%CI)	F1 score (95%CI)	AUROC (95%CI)
KNN	Train	0.77 (0.70, 0.84)	0.81 (0.72, 0.89)	0.78 (0.71, 0.85)	0.86 (0.71, 0.92)
	Test	0.55 (0.50, 0.69)	0.60 (0.56, 0.80)	0.58 (0.47, 0.74)	0.61 (0.47, 0.74)
Naive Bayes	Train	0.55 (0.47, 0.62)	0.97 (0.92, 0.99)	0.69 (0.62, 0.76)	0.68 (0.62, 0.76)
	Test	0.54 (0.48, 0.71)	0.95 (0.83, 1.00)	0.71 (0.58, 0.83)	0.68 (0.58, 0.83)
LR	Train	0.66 (0.59, 0.74)	0.77 (0.68, 0.86)	0.70 (0.62, 0.77)	0.70 (0.62, 0.77)
	Test	0.62 (0.48, 0.76)	0.78 (0.64, 0.90)	0.69 (0.58, 0.82)	0.66 (0.52, 0.82)
Decision Tree	Train	0.67 (0.59, 0.74)	0.83 (0.74, 0.90)	0.72 (0.65, 0.79)	0.67 (0.64, 0.71)
	Test	0.60 (0.47, 0.74)	0.90 (0.75, 1.00)	0.68 (0.52, 0.81)	0.61 (0.52, 0.81)
RF	Train	0.78 (0.72, 0.84)	0.90 (0.83, 0.96)	0.81 (0.75, 0.87)	0.92 (0.85, 0.97)
	Test	0.70 (0.62, 0.74)	0.95 (0.82, 1.00)	0.72 (0.68, 0.84)	0.78 (0.66, 0.84)
XGBoost	Train	0.91 (0.87, 0.95)	0.89 (0.82, 0.95)	0.91 (0.86, 0.95)	0.96 (0.86, 0.98)
	Test	0.64 (0.55, 0.79)	0.63 (0.53, 0.82)	0.67 (0.49, 0.74)	0.65 (0.5, 0.81)
SVM	Train	0.67 (0.59, 0.74)	0.83 (0.74, 0.90)	0.72 (0.64, 0.79)	0.67 (0.64, 0.79)
	Test	0.60 (0.45, 0.74)	0.90 (0.75, 1.00)	0.68 (0.52, 0.81)	0.63 (0.52, 0.81)

KNN, k nearest neighbor; LR, logistic regression; RF, Random Forest; SVM, support vector machine; AUROC, area under the receiver operating characteristics curve.

### 3.4 Interpretable analysis of machine learning models

**Figure 6:**
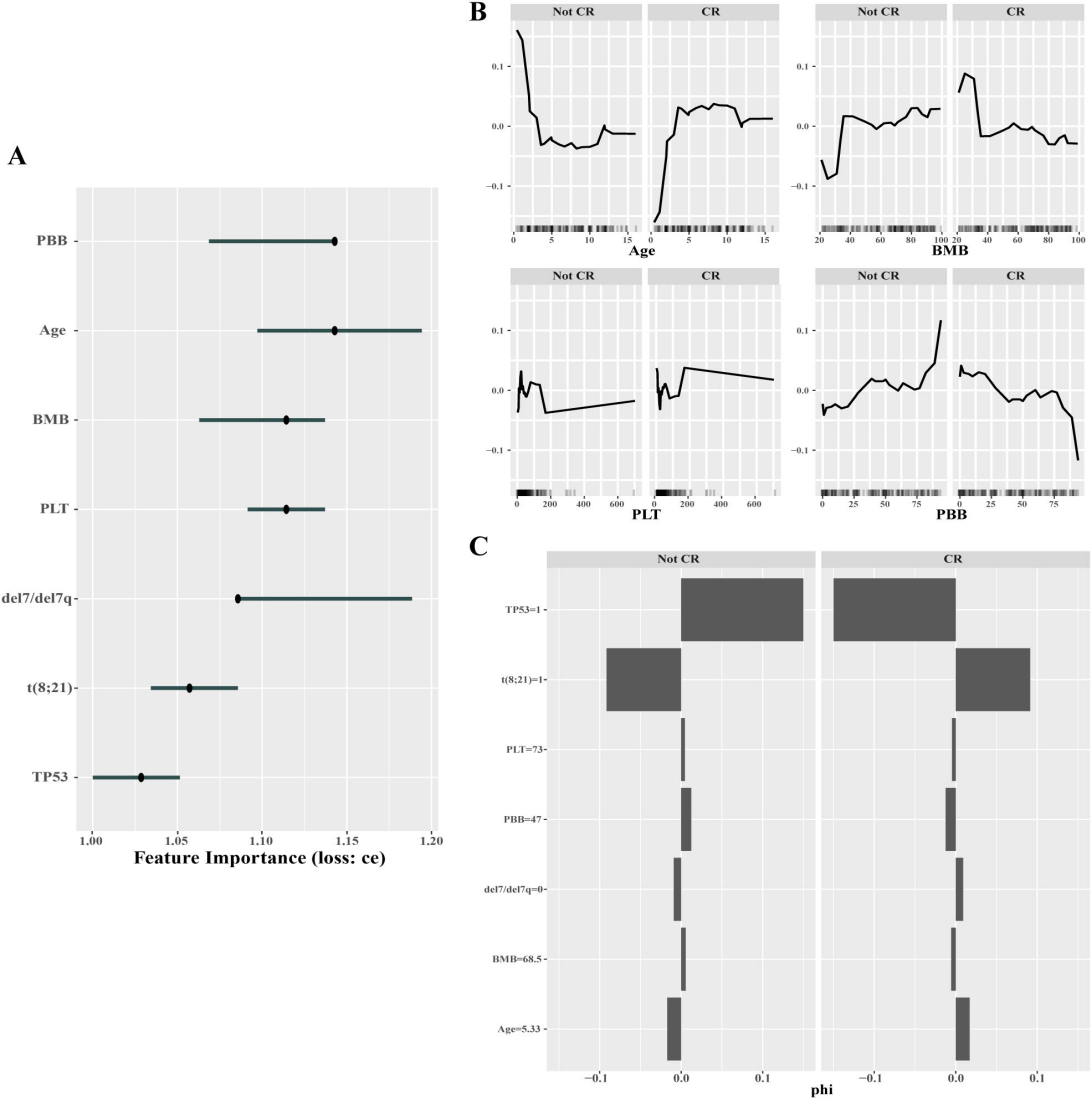
Results of interpretable analysis for RF model in CR prediction. Feature Importance (Feature Imp) **(A)**, Accumulated Local Effect (ALE) plots **(B)** and SHAP (Shapley Additive Explanations) plots **(C)** were employed to interpret the model’s decision pathways and quantify its feature importance. The presence and absence of the t (8;21), del7/del7q and mutation of TP53 are indicated by a value of 1 or 0, respectively. BMB: bone marrow blasts. PBB: peripheral blood blasts.

The machine learning models conducted in our research were black-box models. In order to provide explanations for the decisions made by these models, we use the interpretable machine learning (iml) R package for examination. According to our above results, RF demonstrated the most effective performance in predicting CR. Therefore, we provided an explanation for this model. Firstly, the importance of each feature was computed by the tool called “Feature Imp” within iml package. Feature Imp calculated the increase in the model’s prediction error following the permutation of each feature, providing valuable insights into the importance of individual features in the predictive performance of the model. As depicted in **Figure 6A**, the importance rank for RF model was peripheral blood blasts (PBB), age, bone marrow blasts (BMB), PLT, del7/del7q, t (8;21) and TP53. Subsequently, the Feature Effects were showed by the Accumulated Local Effect (ALE) plots. In the ALE plots, the zero on the vertical axis represents the model average. Positive ALE values signify predictions above the model average, while negative ALE values indicate predictions below the model average. As shown in **Figure 6B**, the continuous variables such as age, BMB, PLT and PBB offered meaningful interpretable information in the data set. Notably, the ALE plots illustrated that the children of older age, less rate of BMB and PBB, and higher level of PLT had greater likelihood of achieving CR. The SHAP (Shapley Additive Explanations) plots showed the features contribution for single predictions with the Shapley value, which help us to understand the machine learning model’s prediction process. As the case showed in **Figure 6C**, the presence of t (8;21), the absence of del7/del7q and age of 5.33 years positively support for the CR prediction, while the presence of TP53 mutation and PBB of 47% exhibited negative associations with the CR prediction. However, the PLT of 73 ×10^9^/L and BMB of 68.5% displayed a lesser contribution to the prediction in this particular case. The interpretation of the impact of these features, as revealed through the ALE plots and SHAP plots, roughly consistent with findings from previous studies and the perceptions of clinicians.

**Figure 7:**
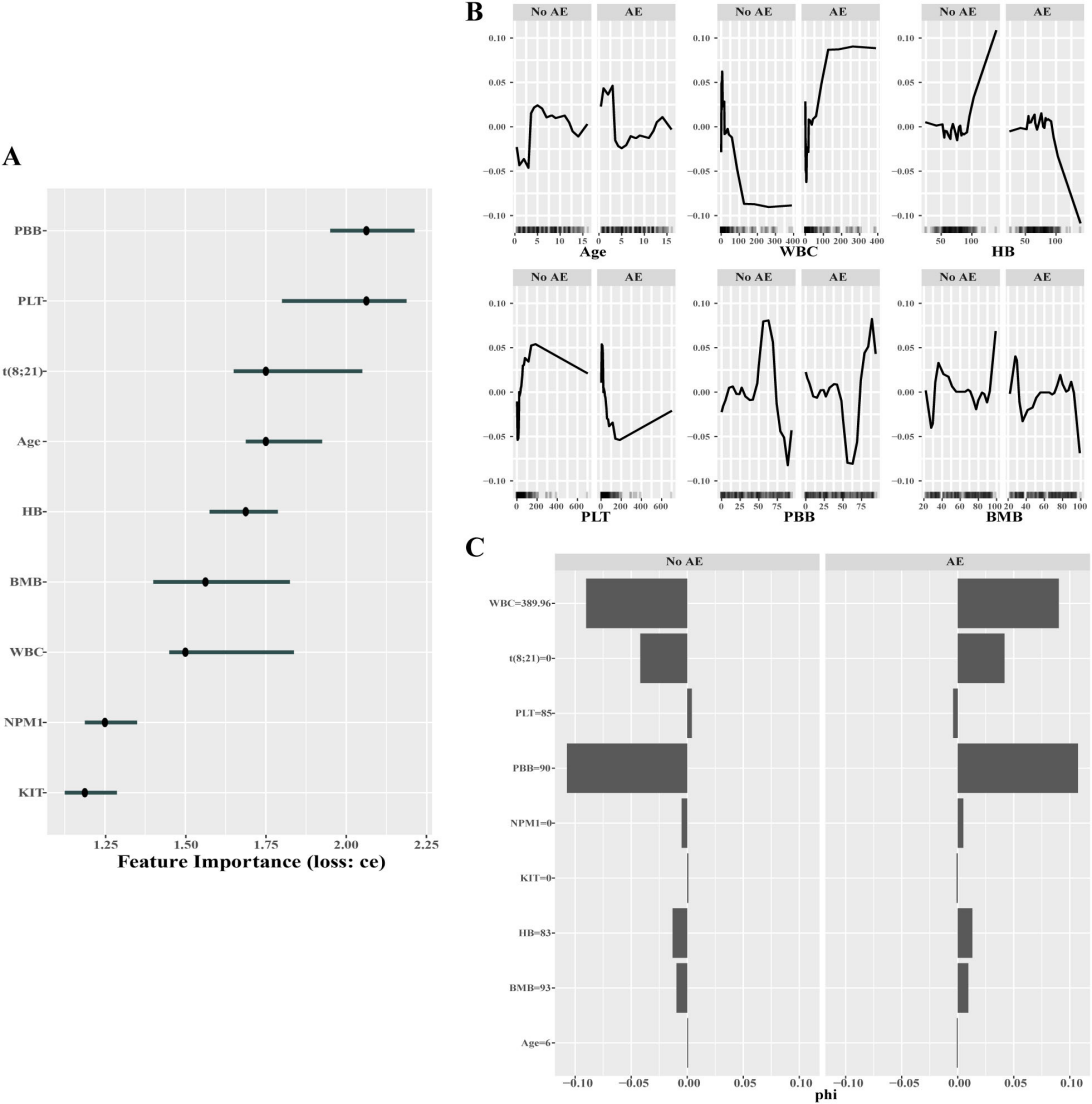
Results of interpretable analysis for RF model in AE prediction. Feature Importance (Feature Imp) **(A)**, Accumulated Local Effect (ALE) plots **(B)** and SHAP (Shapley Additive Explanations) plots **(C)** were employed to interpret the model’s decision pathways and quantify its feature importance. The presence and absence of the t (8;21), NPM1 and KIT are indicated by a value of 1 or 0, respectively. PBB: peripheral blood blasts. BMB: bone marrow blasts.

Similarly, in the prediction of the first adverse event occurrence, the RF model demonstrated the highest performance within our data set. The importance ranking of individual features in the RF model was as follows: peripheral blood blasts (PBB), PLT, t (8;21), age, HB, BMB, WBC, NPM1 and KIT (**Figure 7A**). The ALE plots revealed that younger age, higher level of WBC, PBB, and BMB, and lower levels of HB and PLT were associated with a higher possibility of experiencing adverse event (**Figure 7B**). To enhance the comprehension of the model, we selected a case to compute the Shapley values and generated the SHAP plot. **Figure 7C** illustrates that in this case, with WBC = 389.96, the absence of t (8;21), and PBB = 90, significant Shapley values were observed, indicating the crucial impact of these variables on influencing the model’s prediction.

## 4 Discussion

Currently, the diagnosis of pediatric AML relies on a comprehensive approach including cytomorphology, immunophenotyping, cytogenetics, and molecular genetic analysis (7). These cytogenetic and molecular characteristics are crucial for determining prognosis, risk stratification, and guiding individualized treatment strategies for AML patients (7). However, the rapidly expanding volume of genomic data in AML presents substantial challenges for clinical translation. Machine learning (ML) approaches have demonstrated significant potential for processing complex biomedical datasets and enhancing precision medicine applications in leukemia management (26–28). Although machine learning approaches have been applied to outcome and remission prediction in AML, including adult and pediatric cohorts (29–31), few studies have developed interpretable ML models in pediatric AML using only routinely available baseline clinical parameters to predict induction response and first adverse events. In this study, we constructed seven machine learning algorithms using the clinical data of 206 children diagnosed with AML (excluding APL) at our hospital over a 10-year period. Our analysis revealed ML models, particularly the random forest (RF) algorithm, exhibited excellent predictive performance for both complete remission (CR; AUC 0.90) and adverse event (AE; AUC 0.92).

Our analysis identified platelet count at diagnosis as a significant predictor of both complete remission and first adverse event in pediatric AML. Specifically, pediatric patients with elevated platelet counts (PLT) at diagnosis had both higher CR rates (**Figure 6B**) and reduced incidence of adverse events (**Figure 7B**). A retrospective cohort study conducted in Shanghai involving pediatric AML patients also demonstrated that higher initial platelet counts were associated with improved long-term survival outcomes (32). Furthermore, Zhang et al. (33) reported that AML patients aged 14–60 years with medium platelet counts (50-120×10^9^/L) at diagnosis exhibited significantly longer overall survival (OS) and disease-free survival (DFS) compared to those with either low (< 50×10^9^/L) or high (>120×10^9^/L) platelet counts.

In this study, the first adverse event (AE) was defined as a composite endpoint that included failure to achieve CR, relapse, or death. This approach, while incorporating heterogeneity and competing risks among components, was necessary to increase event rates and statistical power given our limited sample size. As a result, the model should be interpreted as predicting an aggregate “overall treatment failure risk”, rather than providing event-specific probabilities or causal insight into a particular failure mode. In clinical, this model may serve as an early warning indicator to prompt closer surveillance, earlier reassessment, or individualized management; however, it should not be used in isolation to infer which AE component will occur. Consequently, the AE model’s prediction of aggregate risk functions as a practical early warning indicator for severe adverse outcomes. For AE prediction, elevated white blood cell count (WBC) and peripheral blood blast (PBB) percentage emerged as key risk factors (**Figure 7B**), mirroring clinical observations of hyperleukocytosis as a driver of early treatment failure. Higher WBC level can affect the counts of red blood cells and platelets, leading to anemia and an increased risk of bleeding and infection (28). Meanwhile, WBC levels exceeding 100×10^9^/L can result in leucostasis, causing infarction and pulmonary involvement, and an added risk of early morbidity and mortality (34). Recent studies by Bernward et al. (35) have revealed that patients with extremely high levels of WBC (>200×10^9^/L) are more likely to die within the first two weeks after diagnosis compared to patients with lower WBC counts. The early mortality rate in their research can reach up to 30%, markedly exceeding the rates reported by the Berlin-Frankfurt-Münster (BFM) group (17%) (36) and the Children’s Oncology Group (COG) (10.5%) (37). In our study, the mortality rate in patients with very high WBC category is 41.1% (7/17), with an early mortality rate of 17.7% (3/17) within the first two weeks post-diagnosis (the data is not shown). Additionally, Lene et al. (38) identified a WBC count ≥ 100×10^9^/L at diagnosis as an independent risk factor for resistant disease (RD), which caused a substantial proportion of treatment failures in pediatric AML.

To obtain optimal predictive performance in our ML models, we implemented some optimization strategies. Firstly, the selection of features. Feature selection involves identifying the subset of features with the greatest significance for prediction, helping us to construct a model that achieves optimal performance. To determine the most predictive subset of variables in our models, we utilized three methods: random forest, stepwise regression, and joint mutual information maximization (JMIM), and intersected to derive the optimal feature variables for the machine learning algorithms. Additionally, the selection of variables can improve the interpretability of the model. Simultaneously, this selection accelerates the model fitting process and obviates the necessity of gathering numerous costly features. Secondly, the tweak of hyperparameters. For machine learning algorithms requiring hyperparameter tuning, systematic optimization of these parameters offers an effective approach to enhance model performance and predictive accuracy. While machine learning algorithms provide predefined hyperparameter settings, these tunable parameters generally require optimization tailored to individual datasets to attain peak model efficacy (39). In this study, we employed the automated hyperparameter optimization framework within the mlr3 ecosystem to systematically calibrate key algorithms, including k-nearest neighbors (KNN), decision trees, random forests, XGBoost, and support vector machines (SVM), ensuring model configurations were optimized for peak performance across all evaluated datasets. Thirdly, the model interpretation. In our research, interpretable ML techniques from the iml package (e.g., Feature Imp, SHAP, and ALE plots) provided granular insights into model behavior. For example, the SHAP analysis in **Figure 7C** revealed that if the WBC = 389.96, the patient is more likely to experience adverse events. These interpretability tools not only enhanced clinical trust but also validated biological plausibility-a critical step in the translation process.

Our study also has several limitations. First, the research is a single-center design, which means the demographic and treatment heterogeneity across institutions was not captured. Therefore, given the limited sample size of our dataset, the current study is not suitable for the establishment of definitive, clinically actionable cut-off values (e.g., for WBC or age). Our future work should focus on the multi-center validation to calibrate the model’s predictions and determine robust thresholds that can reliably guide clinical intervention. Second, while our models incorporated cytogenetic data, emerging molecular markers (e.g., FLT3-ITD, RAS mutations) (40) were not included due to our modest sample size (n=206), potentially underestimating their prognostic value. Third, our study only focuses on static baseline variables, but overlooks dynamic factors such as minimal residual disease (MRD) status (41), which may refine prediction accuracy. Therefore, further external validation and inclusion of additional molecular data are essential steps before any clinical adoption of our models. Fourth, our sensitivity analysis highlights the potential for optimism bias when relying on a single data partition for validation, a common consideration in smaller cohort studies. Specifically in our research, for the prediction of CR, the RF model achieved a median AUC of 0.94 (95% CI 0.89-0.98) on the training set compared to 0.72 (95% CI 0.65-0.82) on the test set. Similarly, for AE prediction, the median training AUC was 0.97 (95% Cl 0.91-0.99), while the test set AUC was 0.71 (95% CI 0.63-0.81). This variability indicates that training set performance can be optimistic and that test set estimates are sensitive to sampling fluctuations under limited sample size and event counts. Notably, cross-validation in our pipeline was used for hyperparameter tuning within the training set; however, this does not eliminate the inherent optimism of reporting apparent training AUC for the final fitted model. In our future work, we must focus on strategies to improve generalization, such as external validation, simplified models, or incorporation of additional data, and repeated or nested resampling procedures to provide more reliable out-of-sample performance estimates.

## 5 Conclusion

In this research, we evaluated seven machine learner models using our pediatric AML datasets and demonstrated that RF machine learning models based on routine clinical parameters can reliably predict CR and AE in pAML. Additionally, our research may lay the groundwork for more precise risk stratification in pAML.

## Data Availability

The original contributions presented in the study are included in the article/**Supplementary Material**. Further inquiries can be directed to the corresponding authors.
