# Supplementary material for: Development of an interpretable machine learning model to predict complete remission and first adverse event in pediatric acute myeloid leukemia using routine clinical data
Source: Front Oncol. 2026 Apr 10;16:1739432. doi: 10.3389/fonc.2026.1739432 (PMC13105943; doi:10.3389/fonc.2026.1739432)
Supplement: Supplementary file 1 [file DataSheet1.docx]

Supplementary Material

# Supplementary Figures


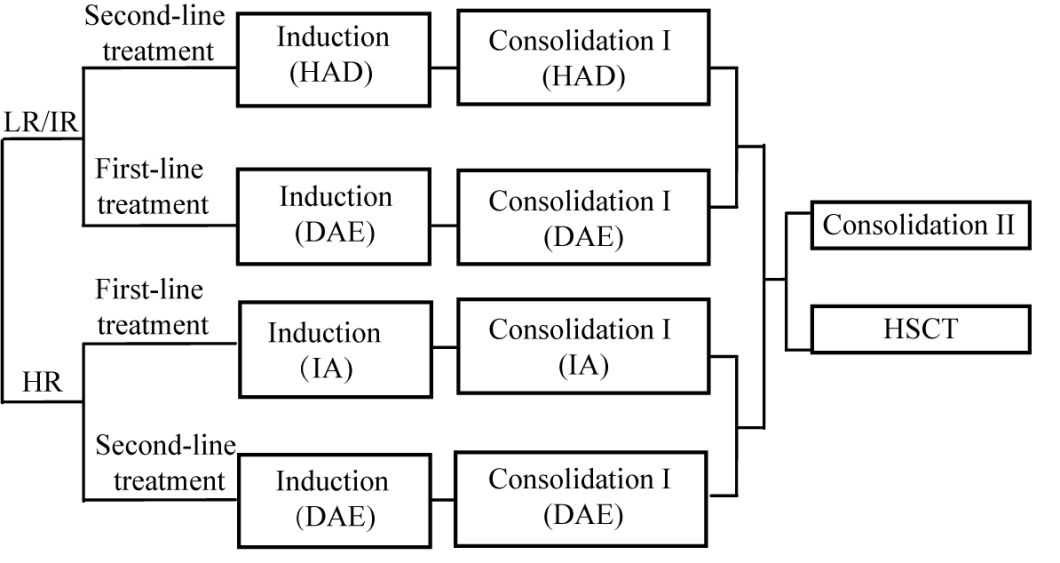


**Supplementary Figure 1.** The AML-2006 protocol developed by our hospital. Induction HAD: homoharringtonine 3mg/m^2^ + cytarabine 200mg/m^2^ + daunorubicin 40mg/m^2^. Induction DAE: daunorubicin 40mg/m^2^ + cytarabine 200 mg/m^2^ + etoposide 100mg/m^2^. Induction IA: idarubicin 10mg/m^2^ + cytarabine 200mg/m^2^. Consolidation I: One additional cycle of the same induction regimen was administered after achieving complete remission (CR). Consolidation II: ① daunorubicin 40mg/m^2^ _+_ cytarabine 2g/m^2^; ② homoharringtonine 3mg/m^2^ + cytarabine 200mg/m^2^; ③ cytarabine 2g/m^2^ _+_ daunorubicin 40mg/m^2^. LR: low risk. IR: intermediate risk. HR: high risk. HSCT: hematopoietic stem cell transplantation.


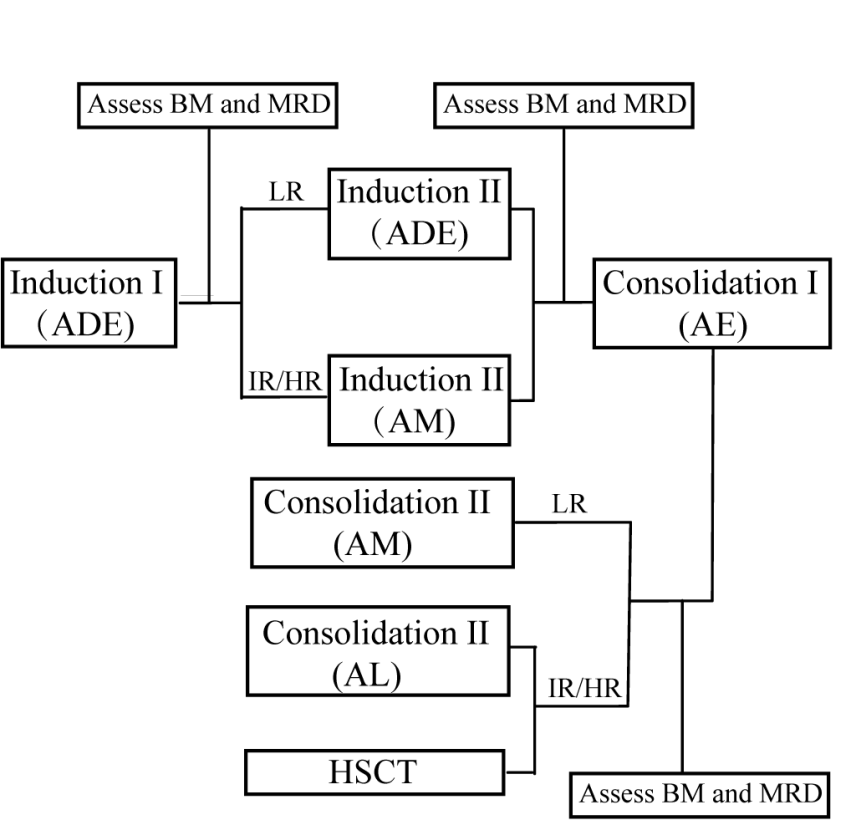


**Supplementary Figure 2.** The 2015-AML-03 protocol developed by our hospital. Induction I (ADE): cytarabine 2g/m^2^ + daunorubicin 150mg/m^2^ + etoposide 500mg/m^2^. Induction II (ADE): cytarabine 1.6g/m^2^ + daunorubicin 150mg/m^2^ + etoposide 500mg/m^2^. Conslidation I (AE): cytarabine 10g/m^2^ + etoposide 750mg/m^2^. Conslidation II (AM): cytarabine 10g/m^2^ + etoposide/idarubicin 48mg/m^2^. Conslidation II (AL): cytarabine 12g/m^2^ + l-asparaginase 12000U/m^2^. BM: bone marrow. MRD: minimal residual disease. LR: low risk. IR: intermediate risk. HR: high risk. HSCT: hematopoietic stem cell transplantation.

^
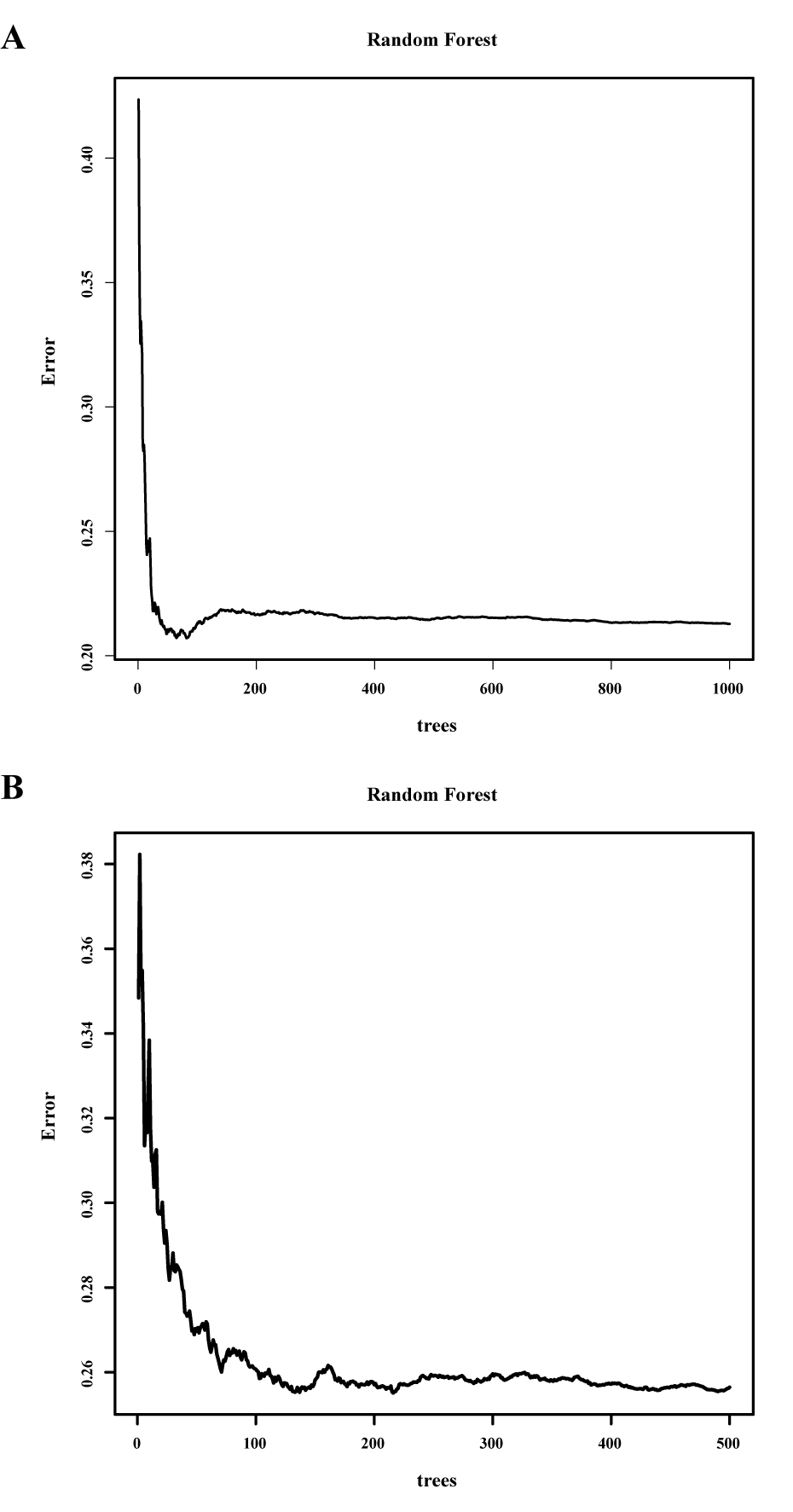
^

**Supplementary Figure 3.** Results of random forest-based feature selection in the two treatment subgroups for CR prediction. **(A)** Feature selection results for AML-2006 treatment subgroup. **(B)** Feature selection results for AML-2015-03 treatment subgroup.

^
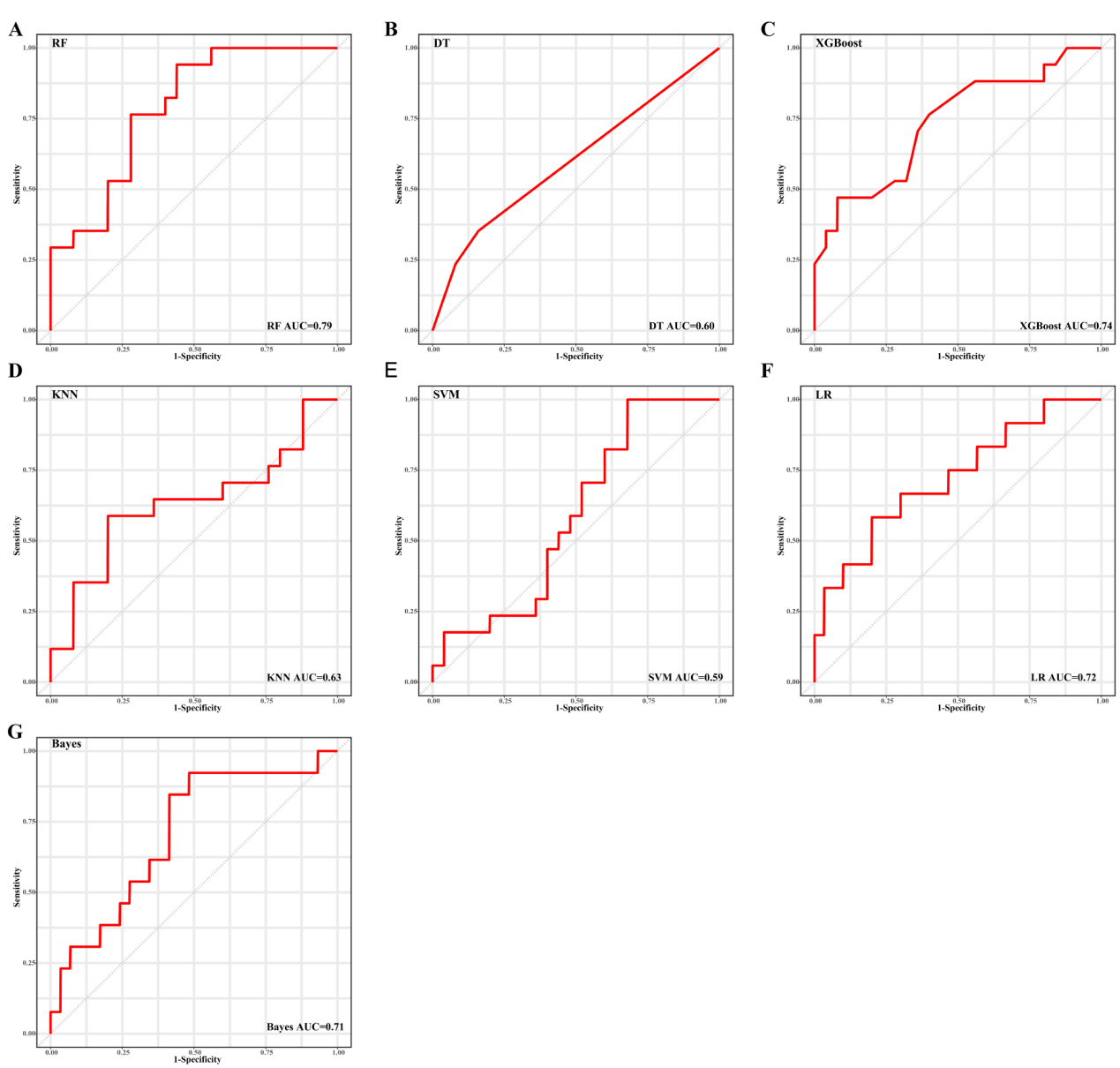
^

**Supplementary Figure 4.** Performance of seven machine learning algorithms for CR prediction, as shown by area under the receiver operating characteristic curve (AUROC) results in the test set. **(A)** random forest (RF), **(B)** decision tree (DT), **(C)** XGBoost, **(D)** k nearest neighbor (KNN), **(E)** support vector machine (SVM), **(F)** logistic regression (LR), and **(G)** Naive Bayes.


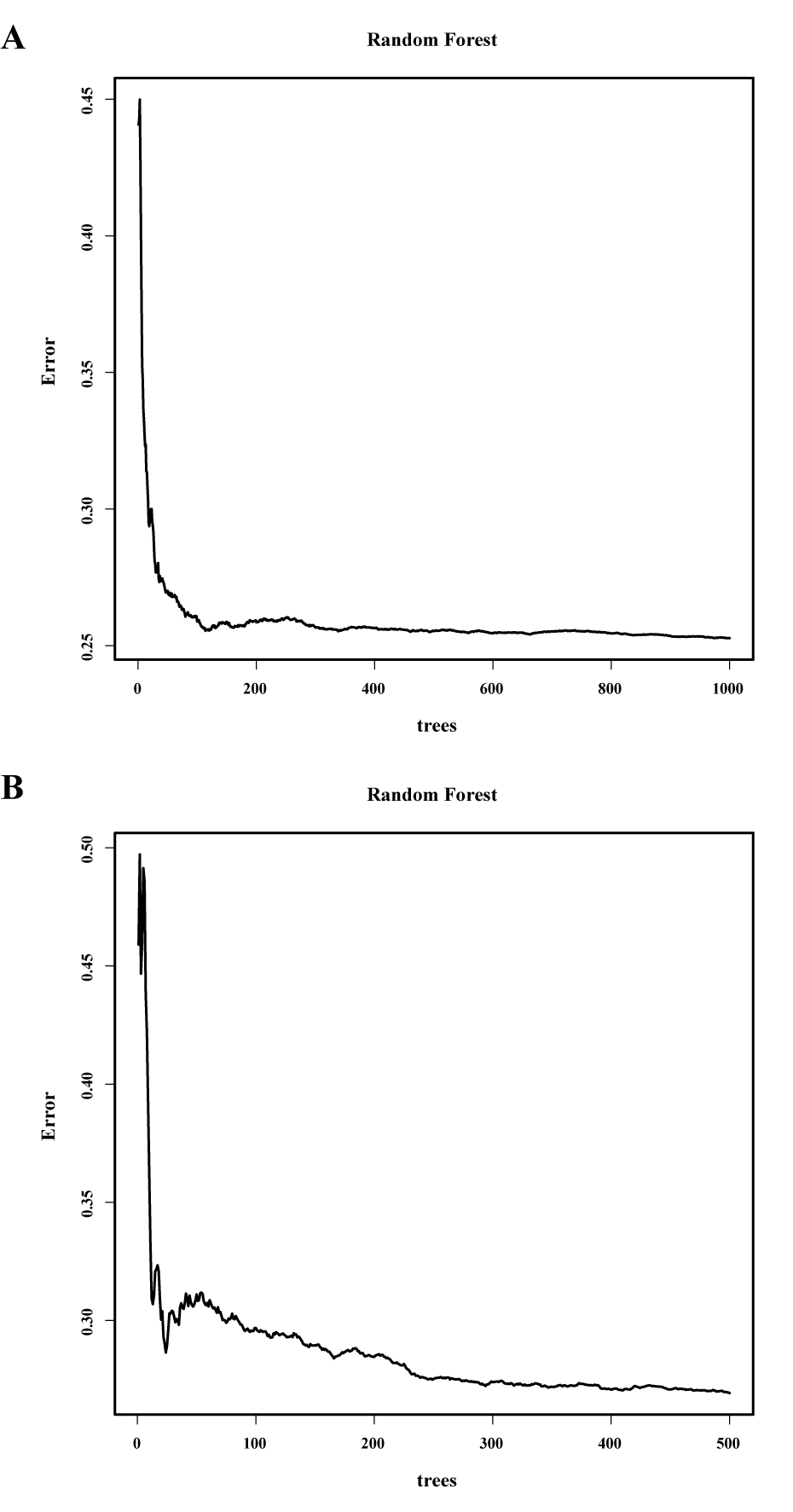


**Supplementary Figure 5.** Results of random forest-based feature selection in the two treatment subgroups for AE prediction. **(A)** Feature selection results for AML-2006 treatment subgroup. **(B)** Feature selection results for AML-2015-03 treatment subgroup.


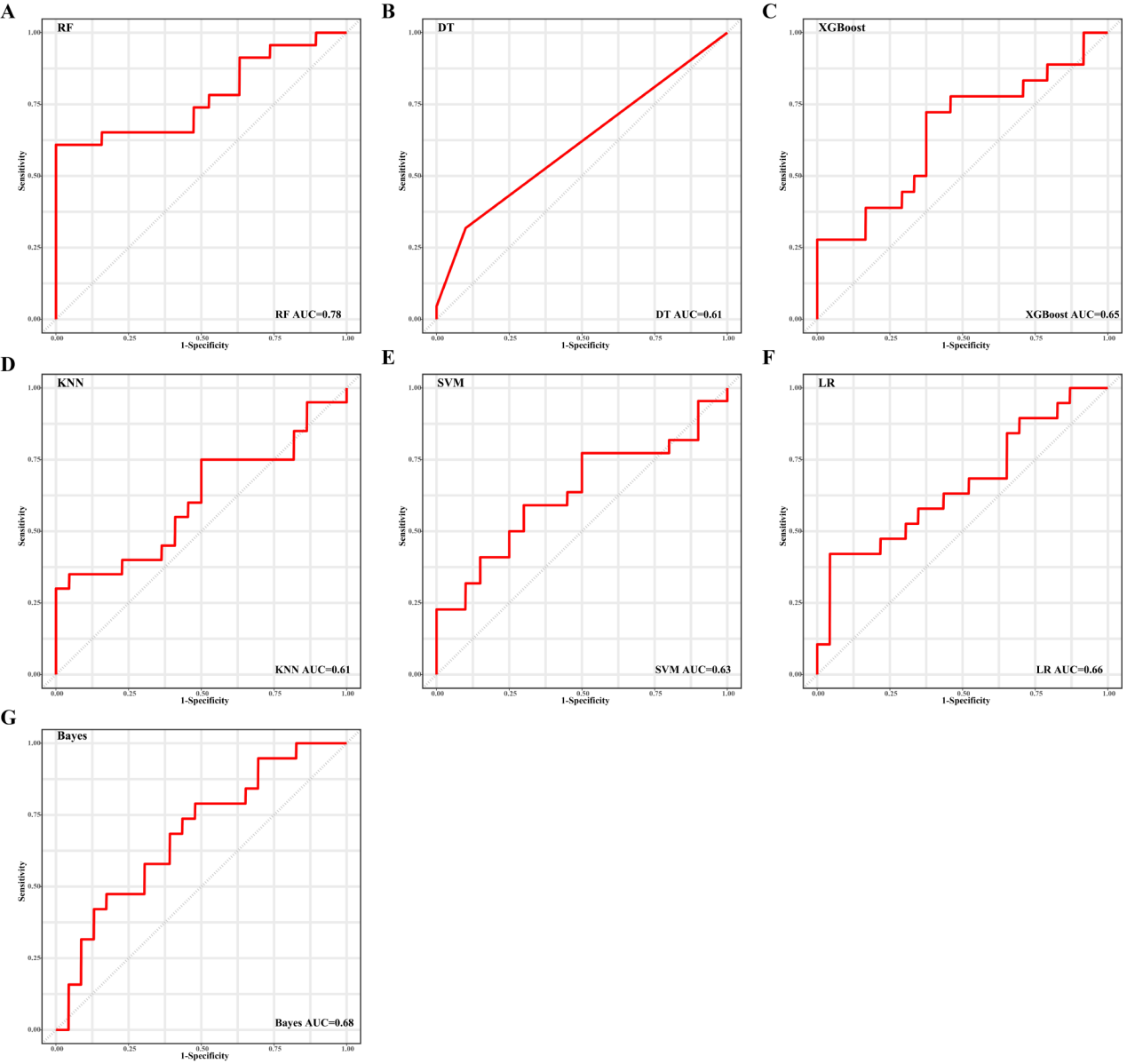


**Supplementary Figure 6.** Performance of seven machine learning algorithms for AE prediction, as shown by area under the receiver operating characteristic curve (AUROC) results in the test set. **(A)** random forest (RF), **(B)** decision tree (DT), **(C)** XGBoost, **(D)** k nearest neighbor (KNN), **(E)** support vector machine (SVM), **(F)** logistic regression (LR), and **(G)** Naive Bayes.

# Supplementary Tables

**Table S1** Molecular genetics and cytogenetics characteristics

| **Cytogenetics** | **Data, N (%)** |
| --- | --- |
| t (10; 11) | 4 (1.94) |
| t (8; 21) | 56 (27.18) |
| t (6; 9) | 3 (1.46) |
| -11/11q- | 8 (3.88) |
| -5/5q- | 4 (1.94) |
| -7/7q- | 14 (6.80) |
| -9/9q- | 19 (9.22) |
| inv (16) | 5 (2.42) |
| t (9; 22) | 1 (0.49) |
| t (9; 11) | 3 (1.46) |
| + 8 | 25 (12.14) |
| + 21 | 13 (6.31) |
| **Molecular genetics** |  |
| ASXL1 | 6 (2.91) |
| BCR-ABL (b3a2) | 1 (0.49) |
| CEBPA | 53 (25.73) |
| CCND2 | 2 (0.97) |
| CBFβ::MYH11 | 5 (2.43) |
| DNMT3A | 8 (3.88) |
| DHX15 | 9 (4.37) |
| EVI1 | 14 (6.80) |
| FLT3-ITD | 13 (6.31) |
| GATA2 | 9 (4.37) |
| HOX11 | 3 (1.46) |
| IDH2 | 3 (1.46) |
| IDH1 | 5 (2.43) |
| KRAS | 3 (1.46) |
| KIT | 21 (10.19) |
| dupMLL | 2 (0.97) |
| MLL-ELL | 1 (0.49) |
| MLL-AF1q | 1 (0.49) |
| MLL-AF10 | 3 (1.46) |
| MLL-AF9 | 5 (2.43) |
| NPM1 | 7 (3.40) |
| NRAS | 27 (13.11) |
| RUNX1 | 5 (2.43) |
| RUNX1::RUNX1T1 | 56 (27.18) |
| TP53 | 7 (3.40) |
| WT1 | 20 (9.71) |

**Table S2** The optimal parameters for each model of CR

| **Model** | **Optimal parameters** |
| --- | --- |
| KNN | k=13 |
| Decision Tree | cp=0.001, minsplit=9, maxdepth=3, minbucket=9 |
| Random Forest | num.threads=1, num.trees=300, mtry=2,  min.node.size=8, max.depth=4 |
| XGBoost | eta = 1, gamma =1, max_depth = 1, nrounds =800,  subsample = 1, eval_metric = "mlogloss" |
| SVM | cost = 3.383855, kernel = "radial", gamma = 6.505882,  type = "C-classification" |

Abbreviations: KNN: k nearest neighbor; SVM: support vector machine

**Table S3** The optimal parameters for each model of adverse events

| **Model** | **Optimal parameters** |
| --- | --- |
| KNN | k=19 |
| Decision Tree | cp=0.05923529, minsplit=7, maxdepth=7, minbucket=3 |
| Random Forest | num.threads=1, num.trees=100, mtry=1,  min.node.size=3, max.depth=7 |
| XGBoost | eta = 1, gamma =5, max_depth = 1, nrounds =800,  subsample = 0.5588235, eval_metric = "mlogloss" |
| SVM | cost = 0.13, kernel = "polynomial", gamma = 10,  type = "C-classification" |

Abbreviations: KNN: k nearest neighbor; SVM: support vector machine
